# IL-33 as a Critical Cytokine for Inflammation and Fibrosis in Inflammatory Bowel Diseases and Pancreatitis

**DOI:** 10.3389/fphys.2021.781012

**Published:** 2021-10-25

**Authors:** Masayuki Kurimoto, Tomohiro Watanabe, Ken Kamata, Kosuke Minaga, Masatoshi Kudo

**Affiliations:** Department of Gastroenterology and Hepatology, Kindai University Faculty of Medicine, Osakasayama, Japan

**Keywords:** fibrosis, IL-33, inflammation, inflammatory bowel diseases, pancreatitis

## Abstract

IL-33 is a pleiotropic cytokine that promotes inflammation and fibrosis. IL-33 is produced by a broad range of cells, including antigen-presenting cells (APCs), epithelial cells, and fibroblasts. IL-33 produced by the innate immune cells has been shown to activate pro-inflammatory T helper type 1 (Th1) and T helper type 2 (Th2) responses. The intestinal barrier and tolerogenic immune responses against commensal microbiota contribute to the maintenance of gut immune homeostasis. Breakdown of tolerogenic responses against commensal microbiota as a result of intestinal barrier dysfunction underlies the immunopathogenesis of inflammatory bowel diseases (IBD) and pancreatitis. Recent studies have provided evidence that IL-33 is an innate immune cytokine that bridges adaptive Th1 and Th2 responses associated with IBD and pancreatitis. In this Mini Review, we discuss the pathogenic roles played by IL-33 in the development of IBD and pancreatitis and consider the potential of this cytokine to be a new therapeutic target.

## Introduction

IL-33 is a nuclear cytokine of the IL-1 family originally discovered as an inducer of classical T helper type 2 (Th2) cells (Cayrol and Girard, 2014; Liew et al., 2016). Similar to other members of the IL-1 family, nuclear pro-IL-33 is cleaved by caspases and secreted in a bioactive form (Afonina et al., 2015). Recent studies have indicated that IL-33 is a pleiotropic cytokine, which not only induces Th2 cells but also activates T helper type 1 (Th1) cells, group 2 innate lymphoid cells (ILC2s), and regulatory T cells (Tregs; Cayrol and Girard, 2014; Liew et al., 2016). IL-33 is released by the innate immune cells, such as epithelial cells and antigen-presenting cells (APCs), upon encountering tissue damage, microbial infection, and allergen exposure (Cayrol and Girard, 2014; Liew et al., 2016). Thus, IL-33 functions as an alarmin cytokine that promotes Th1 and Th2 responses.

IL-33 binds to its transmembrane receptor, suppression of tumorigenicity 2 (ST2) followed by a conformational change leading to interaction between ST2 and IL-1 receptor accessory protein (IL-1RAcP; Liew et al., 2016). Binding of IL-33 to ST2 and IL-1RAcP activates myeloid differentiation primary response 88 (MyD88) to induce nuclear translocation of nuclear factor-kappa B (NF-κB) subunits (Liew et al., 2016).

The immune homeostasis of the gastrointestinal (GI) tract is maintained by the intestinal barrier function and tolerogenic immune responses against intestinal microbiota. It is well established that intestinal barrier dysfunction and excessive immune responses toward intestinal bacteria underlie the immunopathogenesis of inflammatory bowel diseases (IBD) and pancreatitis (Strober and Fuss, 2011; Watanabe et al., 2017a). Disruption of the intestinal barrier and translocation of intestinal bacteria activate APCs, such as macrophages and dendritic cells (DCs), to produce pro-inflammatory cytokines. Sensing of intestinal bacteria by APCs results in robust production of proinflammatory cytokines, leading to pathogenic Th1 and Th2 responses (Takeda and Akira, 2005; Strober and Watanabe, 2011). IL-6, IL-12, IL-23, and TNF-α are prototypical proinflammatory cytokines released by APCs. In fact, the colonic mucosa of patients with IBD is characterized by elevated expression of these cytokines (Strober and Fuss, 2011; Watanabe et al., 2019). Moreover, these proinflammatory cytokine responses have been shown to underlie the immunopathogenesis of pancreatitis (Watanabe et al., 2017a). IL-33, which promotes both Th1 and Th2 responses, has now emerged as a new proinflammatory cytokine promoting the development of IBD and pancreatitis. In this Mini Review, we summarize the key features of IL-33 in terms of its role in IBD and pancreatitis.

## IL-33 and Inflammatory Bowel Diseases

It is well established that the breakdown of tolerogenic immune responses against intestinal bacteria leads to the development of IBD, including Crohn’s disease (CD) and ulcerative colitis (UC; Strober et al., 2006; Strober and Fuss, 2011). Innate immune cells such as macrophages, DCs, and epithelial cells residing in the GI tract produce proinflammatory cytokines and chemokines upon sensing microbe-associated molecular patterns (MAMPs) by pattern recognition receptors. Toll-like receptors (TLRs) and NOD-like receptors (NLRs) are prototypical pattern recognition receptors that sense MAMPs (Takeda and Akira, 2005; Strober et al., 2006). CD and UC are driven by the excessive activation of TLRs and NLRs, followed by the production of proinflammatory cytokines by innate immune cells (Strober and Fuss, 2011). In fact, GI tract mucosa in CD and UC is characterized by elevated expression of IL-6 and TNF-α, which are both produced by macrophages and DCs upon the stimulation with TLR ligands (Watanabe et al., 2019).

Signaling pathways mediated by IL-33 and TLRs share MyD88 as a critical molecule for the activation of downstream transcription factors (Takeda and Akira, 2005; Strober et al., 2006; Liew et al., 2016). Binding of IL-33 to its receptors and recognition of MAMPs by TLRs merge at the level of MyD88 to activate NF-κB and mitogen-activated protein kinases (Takeda and Akira, 2005; Liew et al., 2016). Thus, IL-33 and TLRs might act synergistically to induce proinflammatory cytokine responses, thereby breaking tolerogenic responses against intestinal bacteria. IL-33 is emerging as a pathogenic cytokine responsible for the development of CD and UC.

## IL-33 and Crohn’s Disease

**FIGURE 1:**
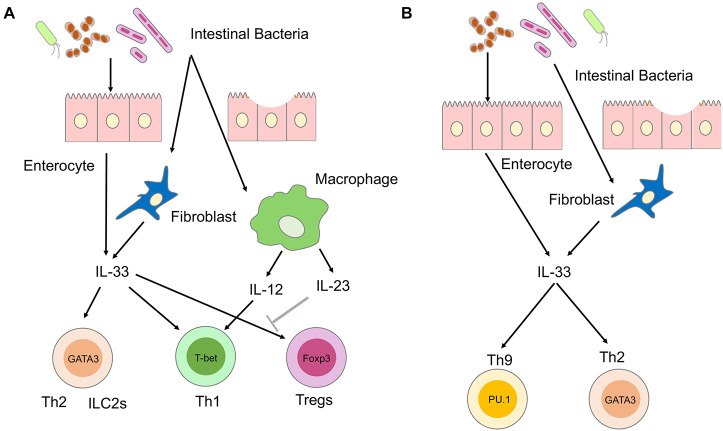
Involvement of IL-33 in the immunopathogenesis of inflammatory bowel diseases (IBD). **(A)** Involvement of IL-33 in the immunopathogenesis of Crohn’s disease (CD). IL-33 is produced by enterocytes or myofibroblasts upon the disruption of the intestinal barrier and exposure to intestinal microbiota. Sensing of intestinal bacteria by macrophages and dendritic cells (DCs) leads to the production of IL-12 and IL-23. IL-33 and IL-12 synergistically activate T helper type 1 (Th1) cells expressing T-bet. IL-33 promotes Th2 responses mediated by Th2 and group 2 innate lymphoid cells (ILC2s), both expressing GATA3. IL-33 is a potent inducer of regulatory T cells (Tregs) expressing forkhead box p3 (FOXP3), and their responses are restrained by IL-23. **(B)** Involvement of IL-33 in the immunopathogenesis of ulcerative colitis (UC). IL-33 is produced by enterocytes or myofibroblasts upon disruption of intestinal barrier and exposure to intestinal microbiota. IL-33 activates Th2 and Th9 cells expressing GATA3 and PU.1, respectively.

Crohn’s disease is a prolonged inflammation of the GI tract characterized by transmural and dense infiltration of lymphocytes and macrophages, presence of granulomas, and submucosal fibrosis (Bouma and Strober, 2003). IL-12, IL-23, and TNF-α produced by macrophages or DCs drive chronic inflammation that characterizes CD by inducing Th1 and Th17 responses. IL-33 produced by myofibroblasts and epithelial cells has been shown to augment Th1 responses associated with CD (Figure 1A; Pastorelli et al., 2010; Bonilla et al., 2012; Sedhom et al., 2013; Baumann et al., 2015; De Salvo et al., 2021). Mice deficient in ST2 are resistant to trinitrobenzene sulfonic acid (TNBS)-induced colitis, an experimental model of CD (Sedhom et al., 2013). IL-33 exacerbates TNBS-induced colitis by disrupting the intestinal barrier function and promoting Th1 responses. Differentiation of Th1 cells is dependent upon IL-12 produced by macrophages and DCs upon the exposure to commensal organisms and TLR ligands (Strober and Fuss, 2011). IL-12 has been shown to act in concert with IL-33 to promote pathogenic Th1 responses (Liew et al., 2016). IL-12 production by macrophages and DCs residing in the submucosa of CD patients is followed by the upregulation of ST2 expression through the activation of signal transducer and activator of transcription 4 (STAT4; Baumann et al., 2015). Thus, IL-33 is involved in the generation of pathogenic Th1 responses associated with CD when commensals infiltrate the damaged intestinal epithelium (Bonilla et al., 2012). This idea is supported by the fact that IL-33 expression levels are increased and correlated with disease activity in CD (Pastorelli et al., 2010; Sedhom et al., 2013).

Massive fibrosis is a prominent feature of CD (Bouma and Strober, 2003). IL-33 activates key players in tissue fibrogenesis, such as Th2 cells and ILC2s (Liew et al., 2016). IL-33 stimulates ILC2 and Th2 cells to produce profibrogenic factors IL-5, IL-13, and TGF-β1 (Liew et al., 2016). IL-33 markedly increased profibrogenic Th2 responses in SAMP1/YitFc mice, which spontaneously develop CD-like ileitis (Pastorelli et al., 2010). IL-33-dependent expansion of ILC2 cells producing IL-5 and IL-13 causes intestinal fibrosis in SAMP1/YitFc mice (De Salvo et al., 2021). These studies suggest that IL-33 produced by epithelial cells and myofibroblasts promotes inflammation and fibrosis associated with Th1 and Th2 responses, respectively.

## IL-33 and Ulcerative Colitis

Ulcerative colitis is a superficial colonic inflammation in which the inflammatory process invariably involves the rectum and extends proximally in a continuous fashion (Bouma and Strober, 2003). The colonic mucosa in UC is characterized by enhanced activation of Th2 and natural killer T (NKT) cells, which produce IL-5 and IL-13 (Strober and Fuss, 2011). In addition to Th2 and NKT cells, recent studies showed that an IL-9-producing subset of helper T cells, called Th9 cells, contributes to the pathogenesis of UC (Gerlach et al., 2014). IL-33 has been shown to be involved in the generation of colitogenic Th2 and Th9 cells (Pastorelli et al., 2010; Sedhom et al., 2013; Hufford and Kaplan, 2014; Figure 1B). Dextran sodium sulfate (DSS)-induced colitis is a well-established model of human UC. ST2-deficient mice were resistant to DSS colitis due to improved recovery from tissue injury, indicating that the IL-33-ST2 axis enhances mucosal healing (Sedhom et al., 2013). Local production of IL-33 by epithelial cells and myofibroblasts also induces activation of Th9 cells leading to the disruption of intestinal barrier function and impairment of tissue-repair mechanisms (Hufford and Kaplan, 2014). In addition, colitogenic cytokine responses, including IL-5 and IL-13, are enhanced by IL-33-mediated activation of ILC2s (Liew et al., 2016). Thus, IL-33 plays a pathogenic role in the development of experimental UC through the disruption of intestinal barrier function and augmentation of Th2 responses. These findings are supported by a clinical observation that in UC patients serum concentrations of IL-33 are higher in the active phase than in the remitted phase (Pastorelli et al., 2010).

In contrast to the above studies showing the pathogenic effects of IL-33, there are also several reports demonstrating the beneficial roles by this cytokine (Oboki et al., 2010; Monticelli et al., 2015). IL-33-deficient mice are sensitive to DSS-induced colitis (Oboki et al., 2010; Monticelli et al., 2015). Monticelli et al. (2015) provided evidence that IL-33 restores intestinal tissue homeostasis by activating ILC2s that produce amphiregulin, a prototypical growth factor for epithelial cells.

## IL-33 and Regulatory T Cells in Inflammatory Bowel Diseases

Accumulating evidence suggest that IL-33 also has a tissue-protective role in experimental IBD. This protective effect of IL-33 is partially mediated by the expansion of gut-associated Tregs expressing forkhead box p3 (FOXP3; Liew et al., 2016). Administration of IL-33 inhibited the development of T cell transfer colitis by the expansion of FOXP3^+^ Tregs (Schiering et al., 2014). Interestingly, IL-23, a pathogenic cytokine in CD, was found to restrain Treg responses through the inhibition of IL-33 signaling pathways (Schiering et al., 2014). Another study also showed that administration of IL-33 ameliorated TNBS colitis through the downregulation and upregulation of Th1 and FOXP3^+^ Treg responses, respectively (Duan et al., 2012). Although these studies support the notion of a protective role played by IL-33 in experimental models of IBD, the relationship between IL-33 and Treg responses has been poorly defined in human IBD. Collectively, IL-33 has been shown to play both pathogenic and beneficial roles in experimental models of IBD, depending on the balance between Th1/Th2/Th9 responses and Treg responses. Further studies are required to determine whether IL-33 is a proinflammatory or anti-inflammatory cytokine in human IBD.

## IL-33 and Pancreatitis

Chronic fibroinflammatory disorders of the pancreas are categorized into chronic pancreatitis (CP) and autoimmune pancreatitis (AIP). CP is a multifactorial disease in which repeated episodes of pancreatic inflammation lead to exocrine and endocrine insufficiency accompanied by fibrosis (Beyer et al., 2020). Genetic or environmental abnormalities that cause intracellular trypsinogen activation underlie CP pathogenesis (Beyer et al., 2020). In contrast, AIP is a unique form of pancreatitis driven by autoimmunity, independent of trypsinogen activation (Stone et al., 2012; Kamisawa et al., 2015; Watanabe et al., 2018). Clinicopathological analysis of a large number of patients with AIP has led to the establishment of a new disease entity, IgG4-related disease (IgG4-RD), and AIP is now considered a pancreatic manifestation of systemic IgG4-RD (Stone et al., 2012; Kamisawa et al., 2015; Watanabe et al., 2018). AIP and IgG4-RD are characterized by enhanced IgG4 Ab responses, storiform fibrosis, and multiple organ involvement (Stone et al., 2012; Kamisawa et al., 2015; Watanabe et al., 2018). Although AIP and CP are completely different disorders of the pancreas, recent studies have highlighted the pathogenic roles played by IL-33 in CP and AIP.

## IL-33 and Chronic Pancreatitis

Excessive activation of trypsinogen by alcohol or high-fat meal is the first step in the inflammatory cascades underlying CP (Watanabe et al., 2017a). Activated trypsinogen causes autodigestion of pancreatic tissues, resulting in the release of damage-associated molecular patterns, which in turn activate the innate immune system to generate proinflammatory cytokine responses, leading to the translocation of intestinal bacteria into the pancreas due to the disruption of the intestinal barrier (Watanabe et al., 2017a). Such translocated bacteria are recognized in the pancreas by nucleotide-binding oligomerization domain 1 (NOD1) expressed in pancreatic acinar cells (Tsuji et al., 2012; Watanabe et al., 2016). NOD1 is an intracellular receptor for small molecules derived from gram-negative intestinal bacteria (Strober et al., 2006). Recognition of bacteria-derived small molecules by NOD1 induces proinflammatory cytokine responses through the activation of NF-κB and type I IFN (Watanabe et al., 2017a). Sensing of commensal bacteria by intracellular NOD1 plays a pathogenic role in the development of experimental CP (see below).

**FIGURE 2:**
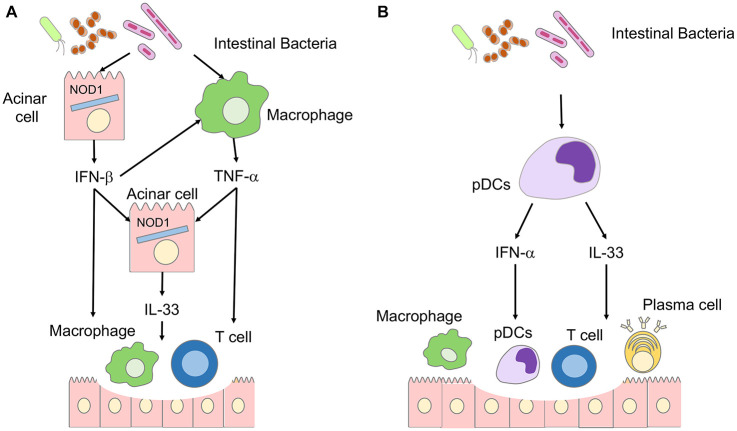
Involvement of IL-33 in the immunopathogenesis of chronic pancreatitis (CP) and autoimmune pancreatitis (AIP). **(A)** Involvement of IL-33 in the immunopathogenesis of CP. Disruption of the intestinal barrier accompanied by pancreatitis induces translocation of intestinal bacteria into the pancreas. Nucleotide-binding oligomerization domain 1 (NOD1) expressed in pancreatic acinar cells recognizes intestinal bacteria. Pancreatic acinar cells produce IFN-β through synergistic activation of signaling pathways mediated by NOD1 and cholecystokinin receptor. Pancreatic macrophages produce TNF-α in response to IFN-β.IL-33 is produced by pancreatic acinar cells in a TNF-α- and IFN-β-dependent manner. The IFN-β-IL-33 axis underlies the immunopathogenesis of CP. **(B)** Involvement of IL-33 in the immunopathogenesis of autoimmune pancreatitis. Intestinal dysbiosis activates pancreatic plasmacytoid dendritic cells (pDCs) producing IFN-α and IL-33. The IFN-α-IL-33 axis underlies the immunopathogenesis of autoimmune pancreatitis.

Repeated injections of cerulein, a cholecystokinin receptor (CCKR) agonist, twice a week for a total of 16–20 times are widely used to induce CP in mice (Watanabe et al., 2017a). Excessive activation of the CCKR-mediated signaling pathways leads to autodigestion of pancreatic tissues through the intrapancreatic activation of trypsinogen (Watanabe et al., 2017a). NOD1-deficient mice are resistant to experimental acute pancreatitis and CP induced by cerulein, indicating that NOD1 is involved in the pathogenesis of pancreatitis induced by CCKR activation (Tsuji et al., 2012; Watanabe et al., 2016). We clarified the molecular mechanism by which NOD1 activation caused CCKR-mediated pancreatitis. We found that NOD1 acted in concert with the CCKR agonist to induce experimental CP (Tsuji et al., 2012; Watanabe et al., 2016, 2017a; Figure 2A). Pancreatic acinar cells secrete IFN-β and C-C motif chemokine ligand 2 (CCL2) as the first mediators of the synergistic activation of NOD1 and CCKR (Watanabe et al., 2016). The subsequent nuclear translocation of NF-κB subunits and STAT3 is required for the production of IFN-β and CCL2 in acinar cells. IFN-β and CCL2, in turn, attract macrophages expressing C-C chemokine receptor type 2 (CCR2) into the pancreas. CCR2^+^ pancreatic macrophages produce TNF-α,another mediator of CP development. IL-33 produced by pancreatic acinar cells in response to IFN-β and IL-33 is the final and third mediator of CP development. The IFN-β-TNF-α-IL-33 axis is required for the generation of chronic fibroinflammatory responses because mice deficient in IFN-β-receptor and those treated with an antibody against ST2 are resistant to the induction of CP (Watanabe et al., 2016).

Chronic pancreatitis is characterized not only by persistent inflammation, but also by massive fibrosis. The IFN-β-TNF-α-IL-33 axis is involved in the process of fibrosis, as shown by the fact that expression of profibrogenic factors such as IL-13 and TGF-β1 is markedly reduced by the blockade of signaling pathways mediated by IFN-β or IL-33. Thus, these data strongly suggest that experimental CP occurs as an IL-33-dependent inflammation resulting from the synergistic interaction between the NOD1 and CCKR signaling pathways. In line with this notion, patients with CP exhibit enhanced expression of IL-33 in acinar cells (Watanabe et al., 2017b). Protective roles played by the IL-33-ST2 pathway have been reported in experimental pancreatitis caused by Coxsackie virus (Sesti-Costa et al., 2013). ST2-deficient mice exhibited enhanced sensitivity to Coxsackievirus-induced pancreatitis through impaired activation of Tregs expressing FOXP3 (Sesti-Costa et al., 2013).

It should be noted, however, that pancreatic expression of IL-33 is not limited to acinar cells. Accumulating evidence suggests that pancreatic stellate cells (PSCs) play a pivotal role in pancreatic fibrosis (Masamune and Shimosegawa, 2013). PSCs exhibit nuclear expression of IL-33 potentiated by proinflammatory cytokines such as TNF-α, IFN-γ,and IL-1β(Masamune et al., 2010). Given that expression of these inflammatory cytokines is elevated in the pancreas during CP, it is possible that PSCs mediate the development of fibrosis in CP in an IL-33-dependent manner.

## IL-33 and Autoimmune Pancreatitis

Repeated intraperitoneal injections of polyinosinic-polycytidylic acid [poly (I:C)] in MRL/MpJ mice lead to the development of AIP accompanied by the destruction of pancreatic acinar architecture, infiltration of immune cells, and fibrosis (Arai et al., 2015; Watanabe et al., 2017b). This experimental AIP is characterized by the accumulation in the pancreas of plasmacytoid dendritic cells (pDCs) that produce large amounts of IFN-α (Arai et al., 2015; Minaga et al., 2020a; Figure 2B). The development of experimental AIP in MRL/MpJ mice requires activation of pDCs producing IFN-α because the depletion of pDCs and the blockade of IFN-α-mediated signaling pathways markedly suppressed chronic fibroinflammatory reactions in experimental AIP (Arai et al., 2015; Minaga et al., 2020a). Based on the observation that the IFN-β-IL-33 axis induces persistent inflammation and fibrosis in CP (Watanabe et al., 2016), we examined the involvement of IL-33 in the development of experimental AIP. Indeed, pancreatic expression of IL-33 was much higher in mice displaying AIP than in control mice (Watanabe et al., 2017b). Depletion and purification experiments utilizing pancreatic mononuclear cells have identified pDCs as a major cellular source of IL-33 (Watanabe et al., 2017b). IL-33 production by pDCs requires production of IFN-α as evidenced by the fact that pancreatic expression of IL-33 was markedly reduced in mice treated with pDC-depleting Ab or neutralizing Ab against the IFN-α receptor (Watanabe et al., 2017b). These studies strongly suggest that pDCs producing both IFN-α and IL-33 accumulate in the pancreas during AIP in an IFN-α-dependent manner. Neutralization of IL-33-signaling pathways by an anti-ST2 Ab attenuated pancreatic inflammation and fibrosis through the downregulation of pDC activation and decreased generation of the profibrogenic factors, IL-13 and TGF-β1 (Watanabe et al., 2017b). Thus, IL-33 produced by pDCs mediates chronic fibroinflammatory reactions in experimental AIP.

Regarding the molecular mechanisms by which pDCs producing IFN-α and IL-33 accumulate in the pancreas of mice displaying AIP, we identified intestinal dysbiosis as a potential trigger (Kamata et al., 2019). Bowel sterilization by antibiotics completely inhibited the development of AIP, which was accompanied by a massive reduction in pancreatic accumulation of pDCs producing IFN-α and IL-33 (Kamata et al., 2019). Cohousing and fecal microbiota transplantation experiments have revealed that intestinal dysbiosis increased sensitivity to experimental AIP *via* pDC activation (Kamata et al., 2019). Collectively, these results support the idea that intestinal dysbiosis leads to the development of experimental AIP through the activation of pDCs producing IFN-α and IL-33.

The clinical relevance of the data obtained in animal studies has been confirmed in human samples. pDCs producing IFN-α and IL-33 are present in the pancreas of patients with AIP and IgG4-RD (Arai et al., 2015; Watanabe et al., 2017b). More importantly, serum concentrations of IFN-α and IL-33 have been identified as novel biomarkers for AIP and IgG4-RD (Minaga et al., 2020b). Patients with AIP and IgG4-RD exhibit higher concentrations of IFN-α and IL-33 than patients with CP and healthy controls (Minaga et al., 2020b). The induction of remission by prednisolone was accompanied by a marked reduction in serum concentrations of these cytokines (Minaga et al., 2020b). Collectively, these data suggest that IL-33 produced by pDCs is involved in the immunopathogenesis of experimental AIP and human IgG4-RD. It should be noted, however, that IL-33 produced by other cells might mediate chronic fibroinflammatory responses in AIP (Furukawa et al., 2017; Ishiguro et al., 2020). M2 macrophages have been identified as IL-33 producers in the salivary glands of patients with IgG4-RD (Furukawa et al., 2017; Ishiguro et al., 2020).

Storiform fibrosis is a prominent feature of AIP (Stone et al., 2012; Kamisawa et al., 2015; Watanabe et al., 2018). As mentioned above, the nuclear expression of IL-33 is observed in PSCs (Masamune et al., 2010). It is possible that PSCs mediate the development of storiform fibrosis in AIP through IL-33-mediated signaling pathways.

## Conclusion

IL-33 is emerging as a pathogenic cytokine in IBD and pancreatitis. IL-33, produced by enterocytes and myofibroblasts, promotes the generation of pathogenic helper T cell responses in IBD. IL-33 produced by pancreatic acinar cells and pDCs contributes to the development of chronic fibroinflammatory responses in CP and AIP, respectively. Although cellular sources of IL-33 are different in IBD and pancreatitis, IL-33 mediates tissue fibrosis in both disorders through induction of pro-fibrogenic factors such as IL-13. Moreover, pancreatitis sometimes develops as an extraintestinal manifestation of IBD (Rogler et al., 2021). IL-33 might be one of pathogenic cytokines accounting for simultaneous occurrence of colitis and pancreatitis though further studies are required. Thus, IL-33 is an attractive therapeutic target for IBD and pancreatitis. However, we have to be cautious regarding the clinical application of IL-33, because this cytokine has immunomodulatory roles, partially through the induction of Tregs.

## Author Contributions

MKr and TW drafted the manuscript and prepared the figures. KM and KK reviewed the manuscript for intellectual content. KM, TW, and MKd were responsible for revising the manuscript. All authors contributed to the study and have approved the final manuscript.

## Conflict of Interest

The authors declare that the research was conducted in the absence of any commercial or financial relationships that could be construed as a potential conflict of interest.

## Publisher’s Note

All claims expressed in this article are solely those of the authors and do not necessarily represent those of their affiliated organizations, or those of the publisher, the editors and the reviewers. Any product that may be evaluated in this article, or claim that may be made by its manufacturer, is not guaranteed or endorsed by the publisher.
